# Drinking Behavior, Taste Preferences and Special Beer Perception among Romanian University Students: A Qualitative Assessment Research

**DOI:** 10.3390/ijerph18063307

**Published:** 2021-03-23

**Authors:** Mirela Anamaria Jimborean, Liana Claudia Salanță, Anna Trusek, Carmen Rodica Pop, Maria Tofană, Elena Mudura, Teodora Emilia Coldea, Anca Farcaș, Maria Ilieș, Sergiu Pașca, Alina Uifălean

**Affiliations:** 1 Department of Food Engineering, Faculty of Food Science and Technology, University of Agricultural Sciences and Veterinary Medicine Cluj-Napoca, 400372 Cluj-Napoca, Romania; mirela.jimborean@usamvcluj.ro (M.A.J.); elena.mudura@usamvcluj.ro (E.M.); teodora.coldea@usamvcluj.ro (T.E.C.); 2 Department of Food Science, Faculty of Food Science and Technology, University of Agricultural Sciences and Veterinary Medicine Cluj-Napoca, 400372 Cluj-Napoca, Romania; carmen-rodica.pop@usamvcluj.ro (C.R.P.); maria.tofana@usamvcluj.ro (M.T.); 3 Faculty of Chemistry, Wroclaw University of Science and Technology, Wybrzeze Wyspianskiego 27, 50-370 Wroclaw, Poland; anna.trusek@pwr.edu.pl; 4 Department of Mathematics and Computer Science, Faculty of Pharmacy, “Iuliu Hațieganu” University of Medicine and Pharmacy, Louis Pasteur Street 6, 400349 Cluj-Napoca, Romania; anca.farcas@umfcluj.ro; 5 Department of Proteomics and Metabolomics, MedFuture Research Center for Advanced Medicine, “Iuliu Hațieganu” University of Medicine and Pharmacy, Louis Pasteur Street 4-6, 400349 Cluj-Napoca, Romania; ilies.maria@umfcluj.ro (M.I.); pasca.sergiu123@gmail.com (S.P.); 6 Department of Hematology, Faculty of Pharmacy, “Iuliu Hațieganu” University of Medicine and Pharmacy, Louis Pasteur Street 6, 400349 Cluj-Napoca, Romania; 7 Department of Pharmaceutical Analysis, Faculty of Pharmacy, “Iuliu Hațieganu” University of Medicine and Pharmacy, Louis Pasteur Street 6, 400349 Cluj-Napoca, Romania; alina.uifalean@umfcluj.ro

**Keywords:** alcohol, prevalence, Romanian university students, non-alcoholic beer, low alcohol beer, public health

## Abstract

The transition from adolescence to adulthood can be a challenging period for many students. This period is associated with an increase in alcohol consumption (AC) which can develop a drinking behavior or shape the preferences for certain alcoholic beverages. The purpose of this study was to analyze the AC pattern among Romanian university students, by investigating the association between taste and consumption, including preferences for special beer. A 30-item omnibus-type questionnaire was distributed to undergraduate students and used to gather sociodemographic data, alcohol expectancies, drinking motives and consequences, and special beer consumption. Results showed a statistically significant relationship between the age of first alcohol use and the existence of an alcoholic family member. The main reasons for AC are taste, sensation, relaxation, and socialization. Both female and male students tend to drink occasionally, with a preference for public places. Female students prefer a sweet taste, choosing special beers over the regular ones. The students’ residence may also influence the choice of special beers. Understanding the students’ drinking behavior and taste preferences is essential to create useful strategies to discourage excessive AC. Special beer, a growing segment in the beverage industry, could represent a healthier and safety alternative to AC.

## 1. Introduction

The alcohol consumption (AC) among university students is different between countries, due to cultural differences, family socioeconomic status, level of education, social-activity, health, and religious reasons [1,2,3]. Nowadays, students drink more than earlier generations, with increasing emphasis on binge drinking and drunkenness [4]. The transition from adolescence to adulthood, living apart from parents, with less parental support can be a stressful time for students, often associated with an increase AC, up to dangerous levels [5,6]. Multiple studies have reported heavy AC among university students as an emerging issue, which is often associated with a significant risk of adverse psychological, social, and physical health consequences, including injuries, unplanned sex, academic failure, and alcohol related problems later in life [7,8,9]. Motives for alcohol use differ between genders, so the adverse outcomes [10,11,12].

The sector of special beers: non-alcoholic beers (NAB) and low-alcohol beers (LAB) is one of the fastest growing segments in the beverage industry. Their increased popularity is mostly due to the beers’ rich fruity flavor and refreshing properties. Fruits, fruit juices, by-products, and fruit extracts are often used to give special beer assortments various flavors, tastes, and aroma [13]. Recent studies have shown that special beer gained recognition also due to its potential health benefits, associated with the high content of phenolic antioxidants and the low ethanol content. For special beers, the level of ethanol should be below 2.5% alcohol by volume (ABV), and therefore, the associated health risks should be much lower. Despite the fact that special beers represent a healthier option, the brewing industry faces other challenges, such as ensuring the flavor stability of special beers, which stimulates brewery technological innovations [14]. The drinking motives usually serve as endorsers towards alcohol use, and represent, practically, the gateway through which more distal factors such as alcohol expectancies, genetic factors, and personality features are mediated [15,16].

AC is an ongoing problem and a multifaceted topic to research. As a consequence, data interpretation must be carried out carefully and efficient actions must be taken, if needed. Often, the combined intervention of public health organizations and education specialists is required to create proper strategies for reducing AC among students. For example, problematic drinking might be diminished by targeting the drinking motives behind. Irrespective of the chosen strategy, the first most important step is to have an in-depth understanding of the students’ perceptions, consumption patterns, and preferences.

In this context, the present study had two main objectives. First, we investigated the drinking behavior among students in order to find gender, age, or residence patterns and evaluate the associations between taste and consumption. Second, we assessed how students relate to special beer (NAB and LAB) as an alternative to regular beer and if taste preferences can shape the desire for special beer consumption.

## 2. Materials and Methods

### 2.1. Students Selection and Procedure

The current study was conducted in academic year 2018/2019 at the University of Agricultural Sciences and Veterinary Medicine from Cluj-Napoca (Romania). Undergraduate university students (18–24 years old) were recruited through posters, flyers, and advertisements posted all over the university campus, email messages, personal communications, and social media. Also, the bachelor, master, and PhD students received an email with a short description of the study and a link to the online questionnaire through Google Drive. Eligibility criteria was restricted to participants who had consumed alcohol in the past 12 months, thus abstinent students were excluded from the analysis. A 30-item omnibus-type questionnaire was used to gather sociodemographic data, alcohol expectancies, drinking motives, relevant information about participants’ families, questions about special beer, and items related to health lifestyle/risks and AC. The types of questions that were chosen for the self-report questionnaire included closed and open questions, filter questions, multiple choice (single answer), selection list questions, and free text questions. The questionnaire design is presented in Table 1. The average time to complete the web-based questionnaire was about 30 min. No identifiers such as name, identity number, nor internet protocol address were recorded in order to preserve the anonymity of the participants. The answers of the questionnaire were downloaded in Microsoft Office Excel format from Google Drive.

### 2.2. Ethics

Ethics approval was obtained from the Faculty of Food Science and Technology, University of Agricultural Sciences and Veterinary Medicine Cluj-Napoca, Romania. Students were informed that participation was voluntary and anonymous, no identifying information was collected. Following the link, participants gave their informed consent to participate. No financial or material incentive was provided for participation in the study.

### 2.3. Statistical Analysis

The participants of the study were naturally categorized in drinkers and non-drinkers. All variables were analyzed considering drinkers’ preferences for different types of alcoholic beverages. In order to analyze the existing relationship between targeted groups of data one have used Chi square test (Phi and Cramer’s V). Here the Phi and Cramer’s coefficients were used to measure the strength of an eventual association. Comparisons between two ranked groups were assessed using Mann–Whitney–Wilcoxon rank sum test. Ranked data was represented as median (quartile 1, quartile 3). Interactions between variables was assessed using two-way ANOVA. All the analysis were performed with SPSS 19.0 (Statistical Package for the Social Sciences, version 19.0. New York, NY, USA) and R 4.0.1. The charts were created using Prism (v 6.01, GraphPad Software, San Diego, CA, USA).

## 3. Results and Discussion

### 3.1. Characteristics of the Study Participants

In the present study, a total number of 1054 of records were received. After applying the eligibility criteria, 1044 valid records were considered for analysis and constituted our study group. The gender structure of the respondents is presented in Table 2. With respect to age, the median age of responders was 21 years (ranging 18–24 years). The analyzed sample was relatively homogenous concerning gender distribution (472 males (45%) and 573 females (55%)). Most responders have their residence in the urban area of Romania (70.30%, of which 44.88% male, and 55.11% female). All responders were of Caucasian race.

### 3.2. Drinking Behavior and Taste Preferences

The first objective of our study was to characterize the drinking behavior among students and to determine if age, gender, or residence could be associated with certain consumption patterns.

**Figure 1:**
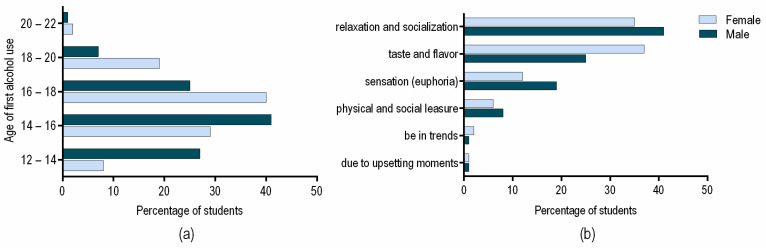
(**a**) The percentages of male and female students related to the age of their first alcohol use; (**b**) The main reasons why male and female students are consuming alcohol.

General drinking habits and behavior investigations within our study unraveled interesting patterns among male and female responders. As presented in Figure 1a, male students start consuming alcohol in their teenage years, half of the respondents beginning to drink before the age of 16. Our results are in agreement with the 2018 World Health Organization (WHO) global report on alcohol use, which states that in many countries of the Americas and Europe, the AC starts before the age of 15 years. Global data are worrying, as 50–70% of 15-year-old students use alcohol, with nearly no difference between male and female [17]. It is suggested that drinking motives in young age may predict drinking patterns later in life [18]. The study conducted by Hingson et al. emphasizes the need to counsel adolescents about alcohol use and to implement efficient policies that delay AC [19]. Another recent study suggests that it would be of interest to focus on the training of specific skills among adolescents. In the case of males, it is probably more useful to train skills such as assertiveness to help to resist peer pressure. For females, it could be interesting to teach other ways of relating successfully with their colleagues, both favoring the creation of social networks with peers who do not drink, such as training in social and assertive skills, and encouraging reinforcing activities that do not involve the use of alcohol [20]. In our study, we observed that for female students, the age of alcohol use onset is postponed to 16–18 years.

When we correlated the age of first alcohol use to the existence of an alcoholic family member, we found a statistically significant relationship (Χ^2^ = 20.64, *p* < 0.001). Adolescents who started to drink at a very young age (12–16 years) were more likely to have had a family member with alcohol problems (Table 3). When treating the age-intervals as ranked data we also observed a statistical significance (*p* < 0.001) with subjects having an alcoholic family member having an earlier age of first drink (alcoholic family members median 14–16 (12–14, 16–18); no alcoholic family members median 16–18 (14–16, 16–18)).

Additionally, there was a statistically significant interaction between gender and the presence of alcoholic family members on the age of first alcohol use (*p* < 0.01, Phi and Cramer’s V coefficients equals 0.15 for females and *p* < 0.05, Phi and Cramer’s V coefficients equals 0.13 for males) indicating a weak association.

The first part of the Table 3 shows the age of the first drink amongst females having or not an alcoholic family member. Most of the girls having an alcoholic family member tend to start drinking between 14–16 years old (36.9%), while for the majority of those not having an alcoholic family member this age is pushed to 16–18 years old (41.9%). Males with alcoholic family member start drinking at 14–16 years old (39.2%), followed closely by even younger age (12–14 years old, 36.0%), while those not having an alcoholic member start drinking alcohol between 14–16 (41.6%).

It is known that individuals with first-degree relatives (mother, father, sister, brother, son, or daughter) with an alcohol problem are two to seven times more likely to develop alcohol problems at some time in their lives than people with nonalcoholic relatives [19,21]. Interestingly, studies including sibling/twin/adopted adolescents aged 12 to 19 years reported that both genetic and environmental factors matter in the development of alcohol use among teenagers, but to a different extent, depending on the time of onset, level of alcohol use, and rates of growth [22,23].

When asked about the reasons for consuming alcohol, more than 70% of the total responders of our study declared that they are consuming alcohol for relaxation, socialization or for its taste and flavor (Figure 1b). Interestingly, most male students drink to relax or socialize, while most female students consume alcohol for the beverages’ taste or flavor. Other studies have also shown that drinking motives can differ between male and female students [24]. Among women, associations between drinking motives and the hourly AC rate were observed, and also, interactions between drinking motives and the impact of the number of friends on the hourly consumption rate [25]. Usually, the most common declared drinking motives for both genders include social, enhancement, coping, or conformity motives. A 2015 study confirms that fun, relaxation, and taste are important motivators for drinking among students. These motives negatively correlate with excessive drinking, while fun was negatively associated with the intention to quit drinking [26]. In turn, if escape, loneliness, social reasons, or recall of alcohol advertising were found to be the main reasons for drinking, a positive association with excessive drinking was identified.

Next, we evaluated the AC frequencies and location among all responders (Table 4).

Concerning the AC frequency, we observed a general tendency for both male and female students to drink occasionally, with a preference for public places, such as restaurants or bars. Our results are in agreement with Zadarko-Domaradzka et al., who found that 70% of the college students in the Carpathian Euroregion (Polish, Slovak, Romanian, and Ukrainian) consume alcohol occasionally [27]. Regarding the consumption location, we obtained a statistically significant difference between genders (Χ^2^ = 9.13, *p* < 0.03), with Phi and Cramer’s V coefficients equals 0.17, showing a weak association. Also, in the case of home, clubs & discos, and public places, a statistically significant relationship between gender and different alcohol frequency was observed (Home: Χ^2^ = 8.05, *p* < 0.05, Phi and Cramer’s V = 0.22; Clubs & Discos: Χ^2^ = 7.78, *p* < 0.05, Phi and Cramer’s V = 0.22; Public places: Χ^2^ = 36.58, *p* < 0.001, Phi and Cramer’s V = 0.22). Therefore, we could observe that there is a significant relationship regarding the frequency of consumption for the women who choose to consume alcohol inside and the men that are choosing the same type of location.

Gender differences remain one of the most reliable determinants of AC. Males drink and are drunk more frequently than females. Males consume more often because of their higher levels of social and enhancement motives, while women due to their higher levels of coping motives and their lower levels of conformity motives [28].

**Figure 2:**
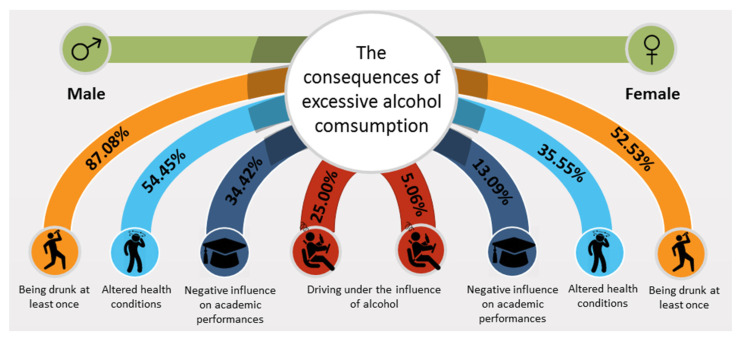
The negative consequences of excessive alcohol consumption among students.

Our results showed that AC was frequently associated with harmful incidence and altered states of health (Figure 2). Worldwide, studies have reported the heavy AC among college student population is associated with numerous negative consequences, such as health issues (vomiting, headache, dizziness, etc.), physical and violence-related problems (assaults, driving under the influence of alcohol, car accidents, etc.), and other injuries [29,30,31]. Women drink to socialize, to relax, to improve their mood, or to escape boredom, while males consume alcohol for image and reputation, they drink “to be cool” and to be more popular among their friends [32,33,34]. The negative consequences of AC are influenced by the body weight, the alcohol tolerance, the speed of consumption, the metabolic rate, the level of hydration, and the food intake [35,36]. Generally, women are more susceptible than men to react to heavy AC due to several reasons. Foremost, women have less enzymes (alcohol dehydrogenase and acetaldehyde dehydrogenase) used to metabolize alcohol, they have a higher body fat to muscle ratio, thus blood and tissue concentrations of alcohol are higher in women, their body size is usually smaller, so women have less water to diffuse the alcohol in their blood stream and, not least, the female hormonal status tends to make women more vulnerable to experience the effects of excessive AC [11]. Men are more likely to take risks while drinking, evidenced by a high rate of car crashes, drink driving over the legal alcohol limit, increased aggressive behaviors, etc. [37,38].

**Figure 3:**
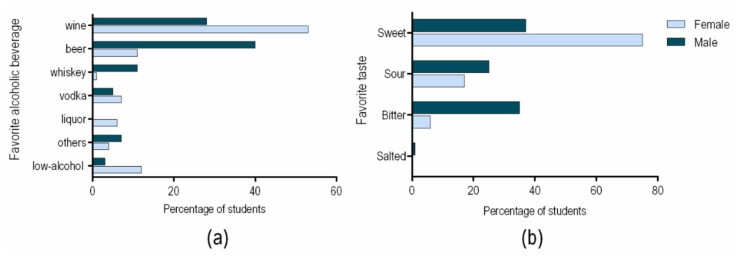
Students’ preferences in terms of alcoholic beverages (**a**) and tastes (**b**).

Regarding the favorite alcoholic beverage among college students, our study shows that beer was preferred by male and wine by female (Figure 3a). When we analyzed the consumption behavior, we found that sensory acceptance was the main selection criteria for all the responders. Taste was one of the main features determining the beverage choices (Figure 3b). The flavor of the alcoholic beverage is an important element in explaining drinking behavior patterns, such as overconsumption. The constant consumption of a specific type of beverage can be related to its chemosensory perception [39].

Concerning the taste preferences, our study revealed that male students tend to prefer bitter drinks, while female students prefer the sweet taste, choosing the special beers over the usual ones. Preference for highly concentrated sweet solutions (sweet liking) has been suggested to be a trait and state marker for alcohol dependence [40]. It is well known that women prefer sweeter products, such as wine or flavored beer, instead of classic beer [41]. The complexity of flavor in terms of the scent, the notes and the structure of the wine are factors that may explain women choices. Moreover, women are interested in a healthy lifestyle, and wine is associated with potential health benefits [42]. Beer is an incredibly versatile beverage, served in various locations such as clubs, bars, and restaurants. Given this context, beer will always be in high demand, particularly for male consumers. Moreover, the men’ preference for beer can be partially attributed to advertising, as in many TV commercials men are the ones consuming beer.

### 3.3. Special Beer: Non-Alcoholic Beer and Low-Alcohol Consumption

Beer is obtained by fermentation of malted barley and it is one of the world’s most popular beverage. A moderate beer consume can have positive effects on health [13,43], due to the bioactive compounds from hops (*Humulus Lupulus* L.), such as: xantohumol, isoxantohumol, humulone, 8-prenylnaringenin and lupulone, used for the bitterness and aroma [44,45]. However, lager beer contains 4–5% volumes ethanol and studies have demonstrated its hepatotoxic effects and its potential to promote different types of cancer [46]. As a reaction, the NAB and LAB market has enjoyed significant growth in the past years, these beers becoming the mainstream option for more and more people.

To gain a more accurate insight with respect to special beer consumption among students, the second objective of our study was to understand how Romanian students relate to special beer (NAB and LAB) as an alternative to regular beer or other alcoholic drinks. Results indicated that gender, age, sociodemographic data might influence the preference for special beers, as summarized in Table 5.

In our study, the percentage of respondents who consumed a special beer at least once was over 90% (93.86% in case of female and 91.80% for male). When asked if they would choose special beers at the expense of high-alcoholic beverages, almost 60% of the respondents responded affirmatively. Of this percentage, more than half was represented by female students (59.58%), who are between 21–24 years old (68.05%) and live in an urban area (65.50%).

As presented in Table 5, our study shows that gender can influence the preference for special beers (Χ^2^ = 13.476, *p* < 0.001, Phi and Cramer’s V coefficients equals 0.12), as female students are more willing to consume this sort of beer. Also, students living in the urban areas of Romania are more likely to choose special beers compared to the students with rural residence (X^2^ = 6.73, *p* < 0.05, Phi and Cramer’s V coefficients equals 0.08). However, the choosing of special beers is not significantly influenced by age.

Because of the differences between males and females considering the reason for consuming alcoholic beverages (especially in the case of the following categories: taste and flavor and sensation (euphoria), relaxation and socialization) we decided to perform subgroup analysis considering these three choices and determining if there was an association in this case between gender and the choice of special beer. There was no association between gender and the choice of special beer in the case of the taste and flavor subgroup (*p* = 0.549), nor in the case of sensation (euphoria) subgroup (*p* = 0.202) or the relaxation and socialization subgroup (*p* = 0.156). This shows that it is possible that the preference in choosing special beers over other alcoholic beverages might be heavily influenced by one person’s reason for drinking the alcoholic beverage, which, in turn is associated with their gender.

When we tested the association between gender, reasons for consuming alcoholic beverages and the preferred taste, we found out that the majority of females preferring the sweet taste are also consuming alcohol because of its taste and flavor. On the other hand, males whose preferences are related to the sweet taste tend to consume alcohol for relaxation and socialization. According to the Chi square test’s values and to the significance value for both males and females (*p* < 0.05), we may confidently conclude that there is a significant relationship between the favorite taste and the reasons for alcohol consumption. 

When asked if they like NAB, 51.86% of responders declared that they do not like it or they have never consumed this type of beer. This high percentage could be explained by the fact that NAB does not deliver a comparable emotional response to the consumers. In order to be accepted by the consumers, a beverage needs to evoke a rich and emotional set of positive associations [47]. While beer and wine are associated with positive emotional responses, the non-alcoholic beer seems to evoke mainly neutral and negative responses such as ration, consciousness, and disappointment [48].

The selection of non-alcoholic versus alcoholic drinks was recently investigated by Blackwell et al., 2020. The results suggested that availability interventions to encourage healthier selection, respectively choosing nonalcoholic rather than alcoholic drinks, may be most effective when changing the relative availability of options, i.e., increasing the proportion of non-alcoholic drinks and consequently decreasing the proportion of available alcoholic drinks [49].

**Figure 4:**
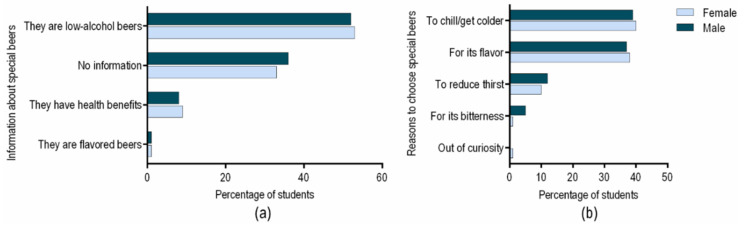
Information about the special beers already knew by students at the time of questionnaire (**a**). The main reasons why male and female students are choosing to drink special beers (**b**).

When asked what information they have regarding special beers, the majority of the respondents were familiar with the low alcohol content of special beer (Figure 4a). However, nearly 40% of students have no information about this beer segment. This student category should be targeted by the awareness AC campaigns. By providing accurate information about the benefits of consuming special beers, students might feel encouraged to consume more special beers, reducing AC. Only a small percentage of responders, around 10%, are aware of the nutritional properties of special beer.

Concerning the health benefits of special beers, Wright et al. have shown that alcoholic beverages, namely wine and beer, are considered healthier than soda or diet soda [50]. This demonstrates that the alcohol content is not the deciding factor when ranking the healthfulness of a beverage. The same study indicated that the simple positioning of the nutritional information on the label of a beverage, even to an alcohol beverage, significantly influenced the consumers perception of its healthfulness. 

**Figure 5:**
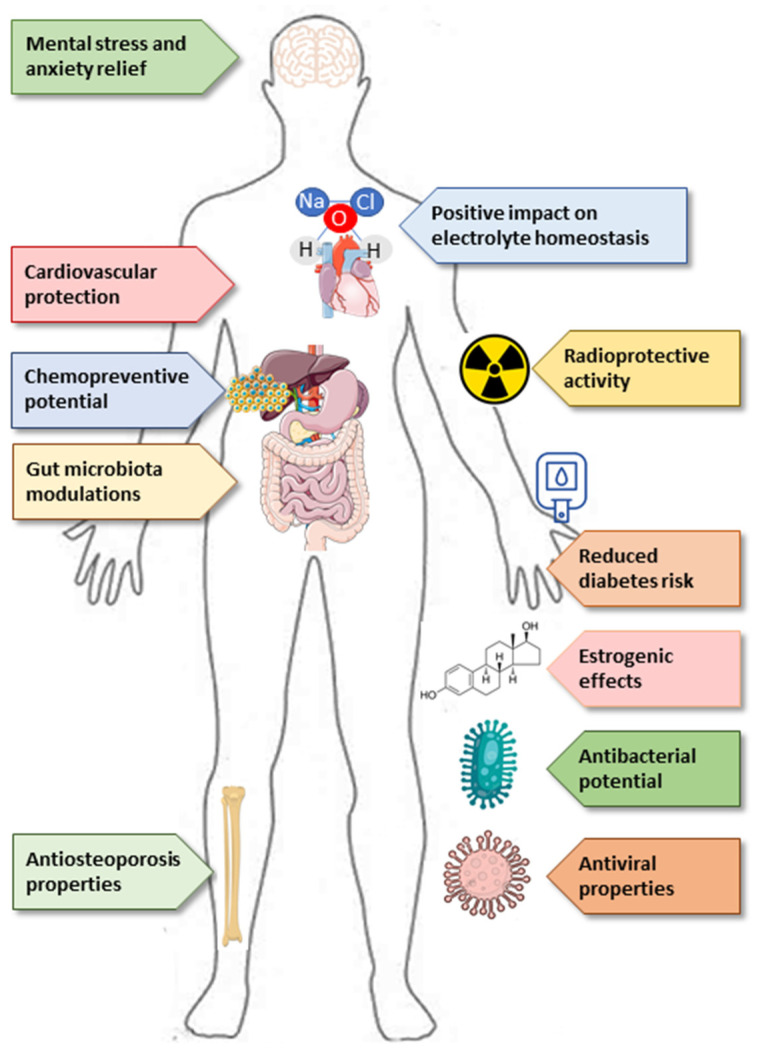
Potential properties of beer’s nonalcoholic fractions [13,51,52,53,54,55,56,57,58,59,60,61].

The functional and potential health properties of special beer have been extensively reviewed in several studies [13,51,52]. While lacking high contents of alcohol, NAB and LAB can be more nutritious and potentially functional than regular beer [13]. NAB and LAB are a reliable source of vitamins, minerals, soluble fibers, polyphenols, and flavonoids [13]. The non-alcoholic beer fraction might improve bone health in postmenopausal women, and the effects of beer on body hydration [53]. When ingested before physical exercises, beer with lower alcohol content has a better rehydration effect, while alcohol-free beer may even have a positive impact on electrolyte homeostasis [54] or gut microbiota [55]. Different studies have focused on the effects of moderate alcoholic and NAB consumption on health and diseases, including cardiovascular disease, obesity, diabetes, cancer, cognitive decline, osteoporosis, with promising results, nevertheless need further particular in-depth investigations [13,56,57]. The consumption of NAB (0.9% ABV) also seems to have a protective action over learning and memory abilities [58]. Franco et al., have examined the effect of NAB on anxiety levels in a stressed population. Subjects rated their stress levels lower after drinking NAB for 14 nights, compared to a control period when they did not drink. All these studies came to the same conclusion: that drinking 330 mL of NAB during evening meals on two weeks may decrease feelings of anxiety and stress. These results are promising, but it must be stressed related research is still at an early stage [59]. A non-alcoholic beer component, β-pseudouridine, was found to be a potent protector against the damage caused by radiation (radioprotective effect) [60,61]. Potential properties of beers’ nonalcoholic fractions are presented in Figure 5.

Finally, we wanted to understand how taste preferences can shape the desire for special beer consumption. Thus, participants of our study were asked about the reasons for choosing a special beer. Most students associated special beer consumption with relaxation, sensory properties and refreshing attributes (Figure 4b). If special beer is chosen for these reasons, public health organizations, along with brewers, could exploit this segment of beer as a powerful instrument to combat heavy AC. Special beer consumption could contribute to reduce alcohol related harm, delivering the same refreshing and sensorial benefits [62].

## 4. Conclusions

The participants in the study were not heavy social drinkers, the majority of students, male and female alike, occasionally reporting AC. Even so, the reported consequences of alcohol intake on health and academic performance were significant. Our results show that AC frequency is related to the contextual influences (places, social contexts, community/friendship, special moments). Most male students start consuming alcohol in their teenage (14–16 years old), while female students are prolonging this period until the first stage of adulthood. We were able to find a statistically significant relationship between the age of first alcohol use and the existence of an alcoholic family member. Concerning the taste preferences, male students tend to prefer bitter drinks, while female students prefer the sweet taste, choosing the special beers over the usual ones. The students’ residence may also influence the choice of special beers consumption over the normal ones.

To our knowledge, this is the first study to assess how students relate to special beer as an alternative to the regular beer and how taste can influence the preference for certain beers. The obtained results can serve and guide the Romanian brewers to improve the aroma quality for special beers, to make them more attractive, especially for young people. In Romania, and also in the EU, various strategies can be applied to improve the special beer acceptability. Increasing the availability of NAB and LAB options in public spaces could ease students to identify alternatives to regular beer. Also, in alcohol awareness campaigns and advertisements, the image of drinking special beers (NAB and LAB) should be promoted as a positive, energetic, and less risky experience in order to reach a larger number of consumers.

## Figures and Tables

**Table 1:** Questionnaire design.

Questions	Explanatory Variables
Q1–Q4	Sociodemographic characteristics: age, gender, the study program, residence;
Q5–Q13	Drinking habits and behaviours: the age of first alcohol use, reasons to drink alcohol, drinking frequency, drinking places, period of highest consumption of alcohol, the existence of an alcoholic family member;
Q14–Q16	Alcoholic taste preferences: the favorite alcoholic beverage, reasons/preferences for alcoholic favorite drink, what tastes appeal most;
Q17–Q20	Risk factors associated with AC: negative consequences, driving under influence of alcohol, drinking problems (altered states of health after drinking episodes, violence-related problems, etc.);
Q21–Q30	Special beer consumption (NAB and LAB): frequency and motives of consumption, sensory preferences, appealing characteristics, expectation attributes.

**Table 2:** Characteristics of the study participants.

	Male		Female	
	N	%	N	%
Eligible respondents	472	45	573	55
Age				
<20	67	14.19	74	12.91
20	76	16.10	102	17.80
21	106	22.67	151	26.35
22	80	16.95	146	25.48
23	66	13.98	57	9.95
24	52	11.02	25	4.36
>24	24	5.08	18	3.14
Residence				
Urban	332	70.33	402	70.15
Rural	140	29.66	171	29.84

**Table 3:** The interaction between gender, age of first drink and alcoholic member.

Gender				Age of First Alcoholic Drink				Total
				12–14	14–16	16–18	18+	
Female	Alcoholic member	Yes	Count	15	38	35	15	103
			% within Alcoholic member	14.6%	36.9%	34.0%	14.6%	100.0%
		No	Count	33	130	197	110	470
			% within Alcoholic member	7.0%	27.7%	41.9%	23.4%	100.0%
	Total		Count	48	168	232	125	573
			% within Alcoholic member	8.4%	29.3%	40.5%	21.8%	100.0%
Male	Alcoholic member	Yes	Count	45	49	24	7	125
			% within Alcoholic member	36.0%	39.2%	19.2%	5.6%	100.0%
		No	Count	82	144	91	29	346
			% within Alcoholic member	23.7%	41.6%	26.3%	8.4%	100.0%
	Total		Count	127	193	115	36	471
			% within Alcoholic member	27.0%	41.0%	24.4%	7.6%	100.0%
Total	Alcoholic member	Yes	Count	60	87	59	22	228
			% within Alcoholic member	26.3%	38.2%	25.9%	9.6%	100.0%
		No	Count	115	274	288	139	816
			% within Alcoholic member	14.1%	33.6%	35.3%	17.0%	100.0%
	Total		Count	175	361	347	161	1044
			% within Alcoholic member	16.8%	34.6%	33.2%	15.4%	100.0%

**Table 4:** The distribution of alcohol consumption frequency and location among male and female students.

Gender	Alcohol Consumption Frequency				Drinking Location	
	Weekly	Once a Month/Once at 2 Months	Occasionally	Others		
Male	20	11	27	5	Home	63
	13	4	41	1	Clubs&Discos	59
	74	13	250	1	Public places	338
	3	0	8	0	Others	11
Female	13	14	57	8	Home	92
	8	14	80	1	Clubs&Discos	103
	27	29	308	5	Public places	369
	4	0	3	2	Others	9

**Table 5:** The preferences for special beers over high alcoholic beverages, classified based on gender, age, and students’ residence, along with the statistical interpretation.

Variables		Preference for Special Beers over High Alcoholic Beverages		*p*-Value
		Total-Yes: 59.96%	Total-No: 40.03%	
Gender				Χ^2^ = 13.476 *p* < 0.001
	Male	40.42%	52.15%	
	Female	59.58%	47.84%	
Age				Χ^2^ = 4.129 *p* > 0.05
	18–20	28.43%	22.52%	
	21–24	68.05%	41.37%	
	>24	3.51%	2.88%	
Residence				Χ^2^ = 6.73 *p* < 0.05
	Rural	29.23%	25.23%	
	Urban	65.50%	65.55%	
	No answer	5.27%	9.09%	

## Data Availability

Not applicable.
